# Supplementary material for: Environmental stress reduces shark residency to coral reefs
Source: Commun Biol. 2024 Sep 9;7:1018. doi: 10.1038/s42003-024-06707-3 (PMC11385207; doi:10.1038/s42003-024-06707-3)
Supplement: Supplementary file 2 — Supporting Information [file 42003_2024_6707_MOESM2_ESM.pdf]

Supplementary Table 1 Confidence set of models following model selection. Presence of a variable or interaction in a model indicated by the + symbol for factors and estimate values for numerical variables. Degrees of freedom (df), small sample size corrected Akaike information Criterion (AICc), ΔAICc and Akaike weights (ω) presented for each model. RI indicates model averaged relative importance for each predictor variable, calculated by summing the Akaike weights for all confidence set models containing them. A value of 1 indicates a variable is very important and occurred in all confidence set models, a value of 0 indicates a variable is unimportant and did not occur in the confidence set models.

Model	Combined environmental SE index (scaled)	Season	Sex	Total Length	Year
30	-0.12	+	+		+
22	-0.12	+			+
**RI**	1.00	1.00	0.60	0.00	1.00

Model	df	logLik	AICc	ΔAICc	ω
368	13	-15045.59	30117.31	0.00	0.67
336	12	-15047.30	30118.72	1.41	0.33

Supplementary Table 2 GLMM results of secondary analysis for residency in grey reef sharks with data from El Niño periods removed following model selection and model averaging. Conditional results are presented. Estimates with unconditional standard error, 97.5% confidence intervals (CI), associated *p* values are presented, with significant results highlighted in bold.

	Estimate	Std. Error	CI	z value	*p* value
Intercept	-2.58	0.29	-3.14, -2.02	-9.24	**<0.001**
**Combined environmental SE index (scaled)**	-0.07	0.01	-0.10, -0.04	-4.73	**<0.001**
Season					
**Wet Season**	-0.35	0.02	-0.40, -0.31	-14.28	**<0.001**
Year					
**2014**	0.34	0.12	0.12, 0.57	2.97	**0.02**
**2016**	0.58	0.17	0.25, 0.91	3.47	**<0.001**
**2017**	0.61	0.17	0.28, 0.94	3.61	**<0.001**
2018	-0.04	0.17	-0.38, 0.29	-2.62	0.79
2019	0.12	0.17	-0.22, 0.46	0.68	0.50
2020	-0.15	0.17	-0.45, 0.20	-0.83	0.41

Supplementary Table 3 Model estimates for pairwise comparisons with 'year' between each 'atoll' in difference in environmental stress exposure. Standard errors, upper (UCL) and lower (LCL) confidence limits, z ratios and p values are provided. Significant results are highlighted in bold.

Pairwise comparisons	estimate	SE	95% LCL	95% UCL	z ratio	p value
2013						
Benares - Blenheim	0.03	0.02	-0.02	0.08	1.66	0.46
Benares - Peros Banhos	-0.03	0.01	-0.07	0.00	-2.58	0.07
Benares - Saloman	-0.02	0.01	-0.06	0.02	-1.58	0.51
Benares - Victory Bank	0.02	0.02	-0.03	0.06	1.01	0.85
**Blenheim - Peros Banhos**	**-0.06**	**0.01**	**-0.10**	**-0.02**	**-4.43**	**<0.01**
**Blenheim - Saloman**	**-0.05**	**0.01**	**-0.09**	**-0.01**	**-3.42**	**0.01**
Blenheim - Victory Bank	-0.01	0.02	-0.06	0.03	-0.85	0.91
Peros Banhos - Saloman	0.01	0.01	-0.01	0.03	1.45	0.60
**Peros Banhos - Victory Bank**	**0.05**	**0.01**	**0.02**	**0.08**	**4.51**	**<0.01**
**Saloman - Victory Bank**	**0.04**	**0.01**	**0.00**	**0.07**	**3.12**	**0.02**
2014						
Benares - Blenheim	0.04	0.02	-0.01	0.09	2.13	0.21
Benares - Peros Banhos	-0.03	0.01	-0.06	0.01	-2.28	0.15
Benares - Saloman	-0.02	0.01	-0.05	0.02	-1.29	0.70
Benares - Victory Bank	0.03	0.02	-0.02	0.07	1.64	0.47
**Blenheim - Peros Banhos**	**-0.07**	**0.01**	**-0.10**	**-0.03**	**-4.83**	**<0.01**
**Blenheim - Saloman**	**-0.06**	**0.01**	**-0.09**	**-0.02**	**-3.76**	**<0.01**
Blenheim - Victory Bank	-0.01	0.02	-0.06	0.03	-0.77	0.94
Peros Banhos - Saloman	0.01	0.01	-0.01	0.03	1.48	0.58
**Peros Banhos - Victory Bank**	**0.05**	**0.01**	**0.03**	**0.08**	**5.17**	**<0.01**
**Saloman - Victory Bank**	**0.04**	**0.01**	**0.01**	**0.07**	**3.67**	**<0.01**
2015						
Benares - Blenheim	0.05	0.02	-0.01	0.10	2.33	0.13
Benares - Peros Banhos	-0.03	0.01	-0.07	0.01	-2.36	0.13
Benares - Saloman	-0.03	0.02	-0.08	0.01	-2.21	0.18
Benares - Victory Bank	0.01	0.02	-0.04	0.05	0.30	1.00
**Blenheim - Peros Banhos**	**-0.08**	**0.02**	**-0.12**	**-0.04**	**-5.11**	**<0.01**
**Blenheim - Saloman**	**-0.08**	**0.02**	**-0.13**	**-0.04**	**-4.81**	**<0.01**
Blenheim - Victory Bank	-0.04	0.02	-0.09	0.01	-2.20	0.18
Peros Banhos - Saloman	0.00	0.01	-0.02	0.02	-0.07	1.00
**Peros Banhos - Victory Bank**	**0.04**	**0.01**	**0.01**	**0.07**	**3.14**	**0.01**
**Saloman - Victory Bank**	**0.04**	**0.01**	**0.00**	**0.08**	**2.86**	**0.03**
2016						
Benares - Blenheim	0.03	0.02	-0.03	0.09	1.50	0.56
Benares - Peros Banhos	-0.04	0.01	-0.07	0.00	-2.59	0.07

Benares - Saloman	-0.02	0.02	-0.06	0.02	-1.36	0.65
**Benares - Victory Bank**	**0.06**	**0.02**	**0.01**	**0.10**	**3.34**	**0.01**
**Blenheim - Peros Banhos**	**-0.07**	**0.02**	**-0.11**	**-0.02**	**-4.15**	**<0.01**
**Blenheim - Saloman**	**-0.05**	**0.02**	**-0.10**	**0.00**	**-3.00**	**0.02**
Blenheim - Victory Bank	0.03	0.02	-0.03	0.08	1.38	0.64
Peros Banhos - Saloman	0.02	0.01	-0.01	0.04	1.88	0.33
**Peros Banhos - Victory Bank**	**0.09**	**0.01**	**0.06**	**0.12**	**8.24**	**<0.01**
**Saloman - Victory Bank**	**0.08**	**0.01**	**0.04**	**0.11**	**6.09**	**<0.01**
2017						
Benares - Blenheim	0.02	0.02	-0.03	0.07	1.22	0.74
Benares - Peros Banhos	0.00	0.01	-0.03	0.03	0.01	1.00
Benares - Saloman	-0.01	0.01	-0.04	0.03	-0.54	0.98
Benares - Victory Bank	0.02	0.02	-0.03	0.06	1.00	0.85
Blenheim - Peros Banhos	-0.02	0.01	-0.06	0.02	-1.55	0.53
Blenheim - Saloman	-0.03	0.02	-0.07	0.01	-1.94	0.29
Blenheim - Victory Bank	-0.01	0.02	-0.05	0.04	-0.39	1.00
Peros Banhos - Saloman	-0.01	0.01	-0.03	0.01	-1.03	0.84
Peros Banhos - Victory Bank	0.02	0.01	-0.01	0.04	1.47	0.59
Saloman - Victory Bank	0.02	0.01	-0.01	0.05	1.94	0.29
2018						
Benares - Blenheim	0.02	0.02	-0.03	0.07	1.05	0.83
Benares - Peros Banhos	-0.01	0.01	-0.04	0.02	-0.68	0.96
Benares - Saloman	-0.01	0.01	-0.05	0.02	-1.10	0.81
Benares - Victory Bank	0.02	0.01	-0.02	0.06	1.66	0.46
Blenheim - Peros Banhos	-0.03	0.01	-0.06	0.01	-1.93	0.30
Blenheim - Saloman	-0.03	0.01	-0.07	0.01	-2.23	0.17
Blenheim - Victory Bank	0.01	0.02	-0.04	0.05	0.37	1.00
Peros Banhos - Saloman	-0.01	0.01	-0.03	0.01	-0.87	0.91
**Peros Banhos - Victory Bank**	**0.03**	**0.01**	**0.01**	**0.06**	**3.29**	**0.01**
**Saloman - Victory Bank**	**0.04**	**0.01**	**0.01**	**0.07**	**3.48**	**<0.01**
2019						
Benares - Blenheim	-0.01	0.02	-0.06	0.05	-0.43	0.99
Benares - Peros Banhos	-0.01	0.01	-0.04	0.03	-0.46	0.99
Benares - Saloman	-0.02	0.01	-0.06	0.02	-1.53	0.54
Benares - Victory Bank	-0.05	0.02	-0.10	0.00	-2.99	0.02
Blenheim - Peros Banhos	0.00	0.02	-0.04	0.05	0.16	1.00
Blenheim - Saloman	-0.01	0.02	-0.06	0.03	-0.77	0.94
Blenheim - Victory Bank	-0.04	0.02	-0.09	0.01	-2.19	0.18
Peros Banhos - Saloman	-0.02	0.01	-0.04	0.01	-2.02	0.26
**Peros Banhos - Victory Bank**	**-0.04**	**0.01**	**-0.08**	**-0.01**	**-3.67**	**<0.01**
Saloman - Victory Bank	-0.03	0.01	-0.07	0.01	-2.18	0.19
2020						

Benares - Blenheim	0.01	0.02	-0.04	0.06	0.49	0.99
Benares - Peros Banhos	0.01	0.01	-0.02	0.05	1.03	0.84
Benares - Saloman	0.00	0.01	-0.04	0.04	0.11	1.00
Benares - Victory Bank	0.00	0.02	-0.05	0.04	-0.18	1.00
Blenheim - Peros Banhos	0.00	0.01	-0.04	0.04	0.25	1.00
Blenheim - Saloman	-0.01	0.02	-0.05	0.04	-0.49	0.99
Blenheim - Victory Bank	-0.01	0.02	-0.06	0.04	-0.67	0.96
Peros Banhos - Saloman	-0.01	0.01	-0.03	0.01	-1.59	0.50
Peros Banhos - Victory Bank	-0.02	0.01	-0.05	0.01	-1.43	0.61
Saloman - Victory Bank	0.00	0.01	-0.04	0.03	-0.36	1.00

Supplementary Table 4 Variables used for the assessment of shallow coral reef stress, including source and temporal and spatial resolution. All information on variable products can be found in the Earth Engine Data Catalogue (https://developers.google.com/earth-engine/datasets/) and Williamson et al. [1].

**Environmental stress variable**	**Temporal resolution (hour)**	**Spatial resolution (km)**	**Start year**	**End year**	**Data source** (https://developers.google.com/earth-engine/datasets/catalog/)
cloud cover	3	11	1978	2021	NOAA Climate Data Record (CDR) of Cloud Properties from AVHRR Pathfinder Atmospheres - Extended (PATMOS-x), Version 5.35.3 [2]
current, salinity	24	9	1992	2023	HYCOM: Hybrid Coordinate Ocean Model, Water Velocity [3]
depth	NA	0.05	2017	2024	Sentinel-2 MSI: MultiSpectral Instrument, Level-2A [4]
SST, DHW, SST anomaly, SST variability	24	4.6	2002	2022	Moderate-resolution Imaging Spectroradiometer (MODIS) Aqua Data [5]
wind	24	4	1981	2022	NOAA AVHRR Pathfinder Version 5.3 Collated Global 4km Sea Surface Temperature[6]

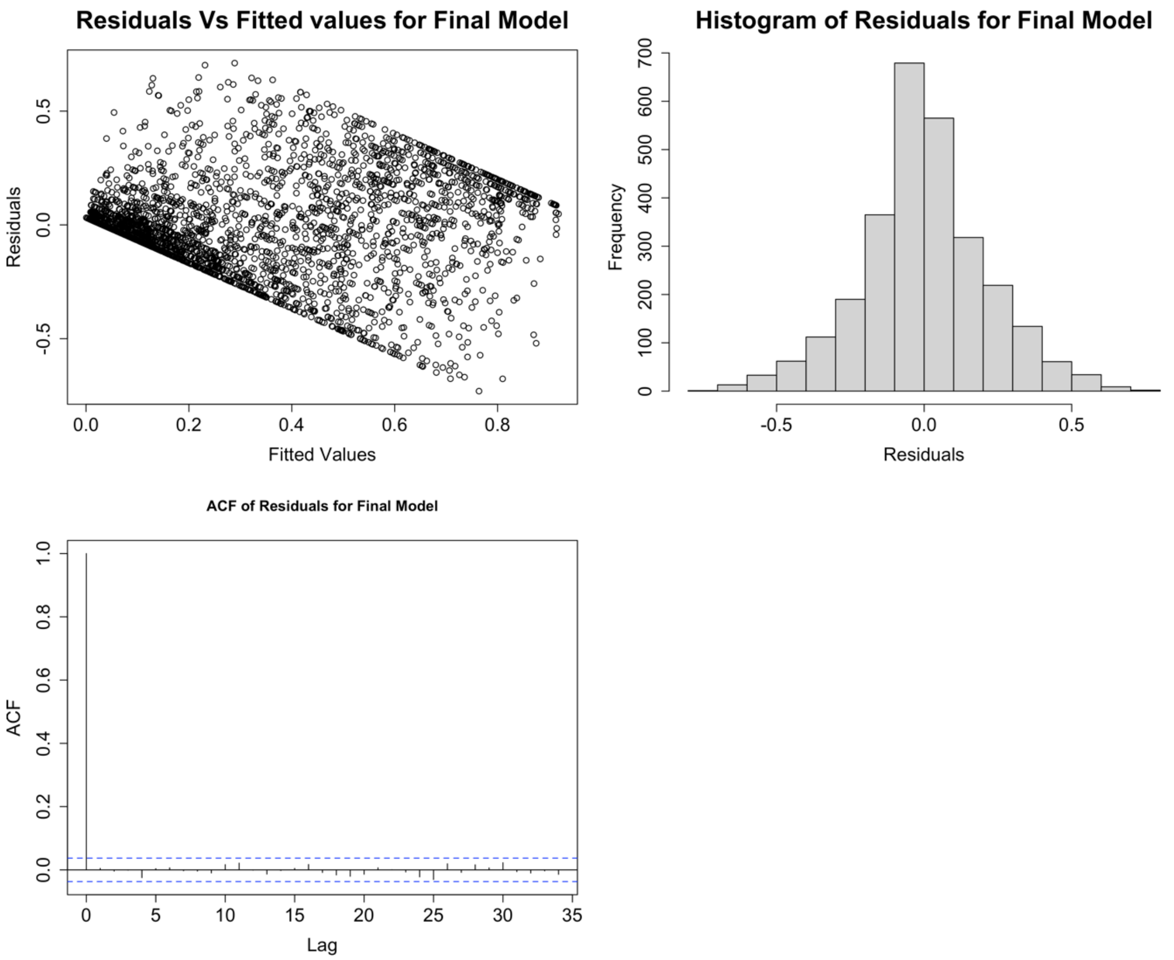

Supplementary Figure 1 Plots of global model residuals to check for heteroscedasticity, autocorrelation and errors checked for binomial distribution. Binomial distribution of residual vs fitted and histogram of residuals indicate lack of homoscedasticity and a binomial distribution. ACF values indicate no autocorrelation.

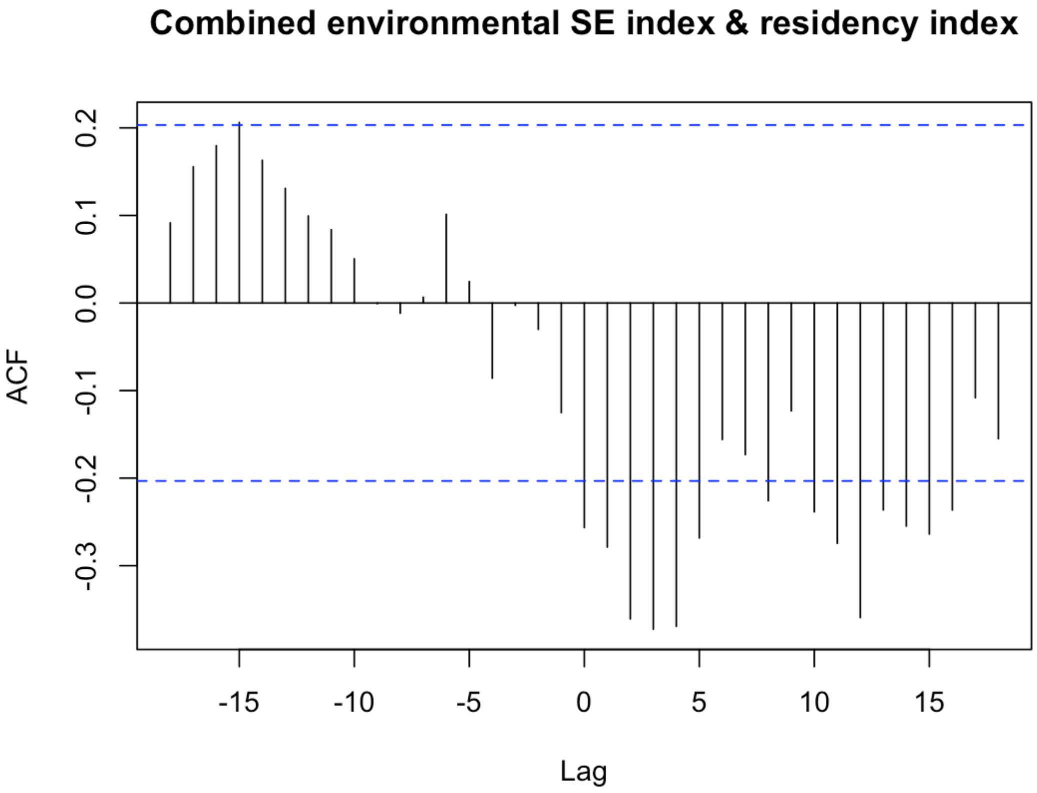

Supplementary Figure 2 A ccf plot with mean combined environmental SE index and mean residency, and lag set at 0.25 confidence intervals are indicated by the blue dotted lines and lag values that cross these lines are deemed to be significant.

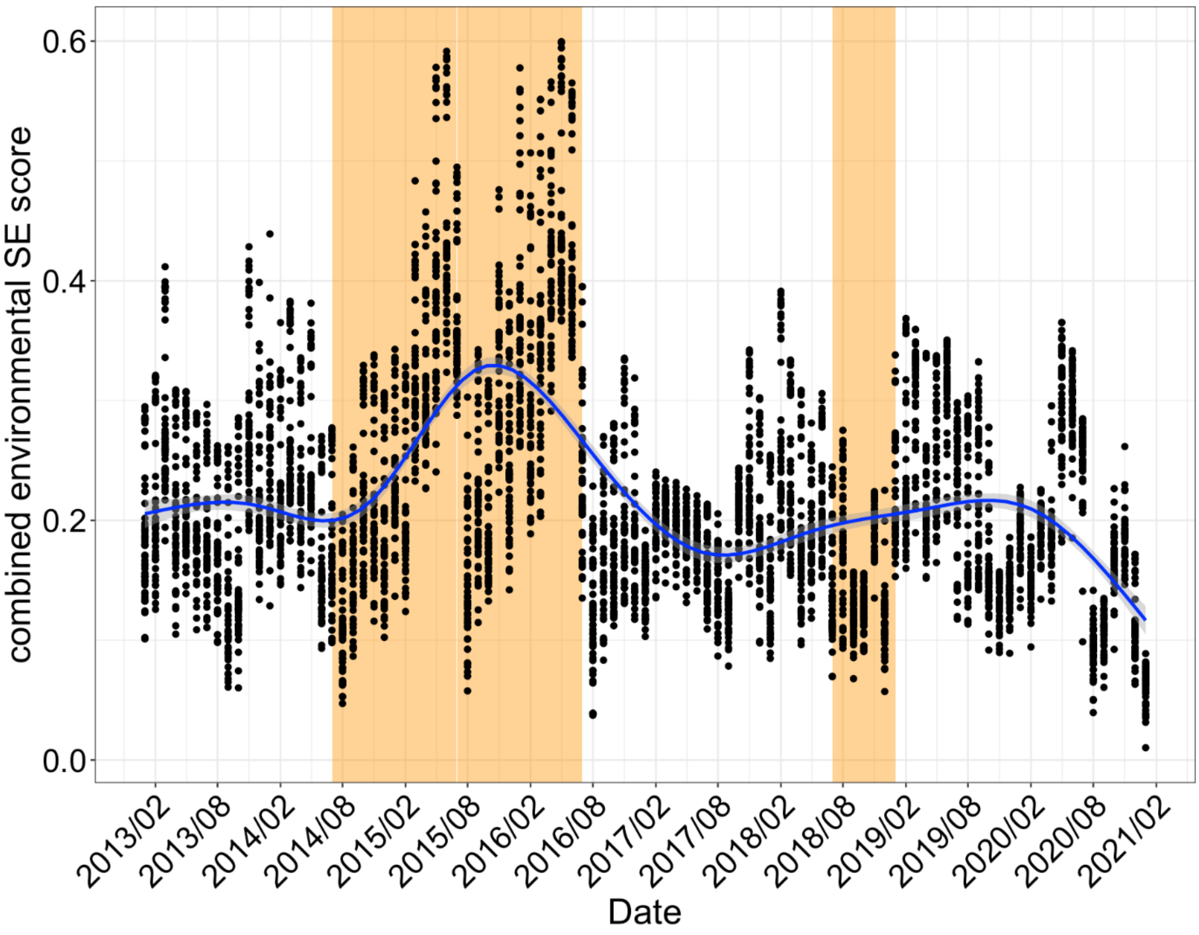

Supplementary Figure 3 Monthly combined environmental SE scores taken at all receivers from 01/03/2013 to 30/11/2020. Trend line in blue with 95% standard error in grey. El Niño events are highlighted in orange.

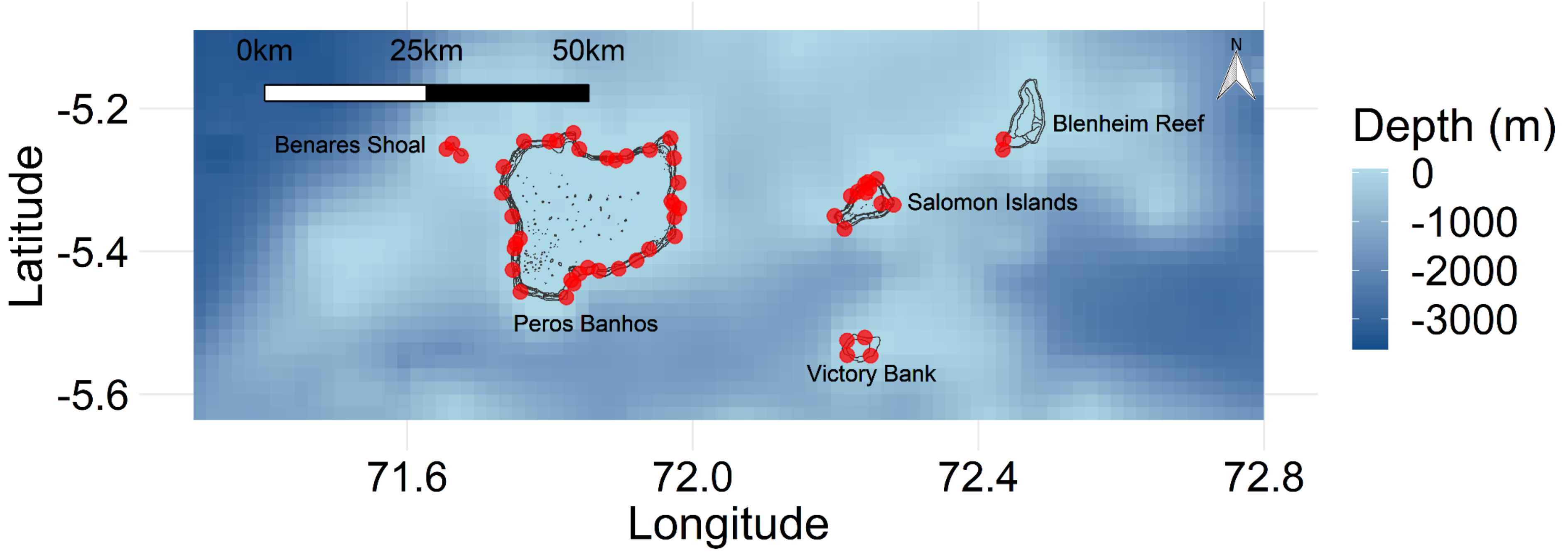

Supplementary Figure 4 Acoustic array in the 5 selected atolls of the Chagos Archipelago with the locations of 54 acoustic receivers, used for data collection, shown in red. Grey lines show the contours of major submerged geographic features. Depth scale, scale bar and north arrow are provided.

Supplementary References

1 Williamson, M. J. *et al.* Monitoring shallow coral reef exposure to environmental stressors using satellite earth observation: the reef environmental stress exposure toolbox (RESET). *Remote Sens Ecol Conserv* **8**, 855-874 (2022). https://doi.org:10.1002/rse2.286

2 Heidinger, A. K., Foster, M. J., Walther, A., Zhao, X. & NOAA CDR Program. NOAA Climate Data Record (CDR) of Cloud Properties from AVHRR Pathfinder Atmospheres - Extended (PATMOS-x), Version 5.3. *NOAA National Centers for Environmental Information.* (2014). https://doi.org:10.7289/V5348HCK

3 Cummings, J. A. & Smedstad, O. M. in *Data assimilation for atmospheric, oceanic and hydrologic applications (Vol. II)* (eds Seon Ki Park & Liang Xu) 303-343 (Springer Berlin Heidelberg, 2013).

4 Li, J. *et al.* Automated global shallow water bathymetry mapping using Google Earth Engine. *Remote Sens* **13**, 1469 (2021). https://doi.org:10.3390/rs13081469

5 NASA Goddard Space Flight Center, Ocean Ecology Laboratory & Group, O. B. P. Moderate-resolution Imaging Spectroradiometer (MODIS) Aqua Remote-Sensing Reflectance Data. 2018 Reprocessing. NASA OB.DAAC, Greenbelt, MD, USA https://doi.org:10.5067/AQUA/MODIS/L3M/RRS/2018

6 Merchant, C. J., Harris, A. R., Roquet, H. & Le Borgne, P. Retrieval characteristics of non-linear sea surface temperature from the Advanced Very High Resolution Radiometer. *Geophys Res Lett* 36 (2009). https://doi.org:10.1029/2009GL039843
