# Supplementary material for: Environmental stress reduces shark residency to coral reefs
Source: Commun Biol. 2024 Sep 9;7:1018. doi: 10.1038/s42003-024-06707-3 (PMC11385207; doi:10.1038/s42003-024-06707-3)
Supplement: Supplementary file 3 — Description of additional supplementary file [file 42003_2024_6707_MOESM3_ESM.pdf]

Description of additional supplementary file.

File name: Supplementary data 1

File description: Metadata of 122 tagged grey reef sharks
